# Renal Artery Occlusion After Standard Endovascular Aneurysm Repair: Incidence, Mechanisms, and Individualized Management

**DOI:** 10.3390/jpm16100519

**Published:** 2026-10-05

**Authors:** Rodolfo Pini, Pietro Fiorio, Betti Shyti, Marcello Lodato, Mohammad Abualhin, Antonio Cappiello, Andrea Vacirca, Paolo Spath, Enrico Gallitto, Gianluca Faggioli, Mauro Gargiulo

**Affiliations:** 1 Vascular Surgery, Department of Medical and Surgical Sciences (DIMEC), University of Bologna, 40138 Bologna, Italybettishyti@gmail.com (B.S.);; 2 Vascular Surgery Unit, IRCCS Sant’Orsola, Azienda Ospedaliero-Universitaria, 40138 Bologna, Italy

**Keywords:** abdominal aortic aneurysm, standard endovascular aneurysm repair, renal artery compromise, renal artery occlusion, renal stenting, acute kidney injury, renal salvage, personalized management

## Abstract

**Background/Objectives:** Direct renal artery compromise after standard endovascular aneurysm repair (EVAR) is rare and may result from mechanical ostial coverage or atheroembolism. We estimated its incidence and described mechanism-specific management and renal outcomes. **Methods:** We retrospectively reviewed all consecutive elective standard infrarenal EVAR procedures performed at a single center from March 2016 to June 2025, excluding repairs requiring planned renal incorporation. The primary event was complete renal artery occlusion or severe compromise requiring revascularization. We assessed mechanism, timing, management, renal function, dialysis, target-vessel patency, and survival. **Results:** Among 973 EVAR procedures, three patients developed renal artery compromise (0.31%; exact 95% confidence interval, 0.06–0.90%): inadvertent right ostial coverage, right atherothrombotic occlusion from a thrombus-bearing proximal aorta, and left occlusion with severe right compromise after proximal cuff deployment. Endovascular salvage succeeded in both attempted cases; the unilateral embolic occlusion was managed conservatively. All patients developed acute kidney injury, but none required dialysis. Median follow-up was 67.6 months. **Conclusions:** Recognized renal artery compromise after standard EVAR was uncommon in this cohort. These three cases illustrate a mechanism-informed approach to individualized assessment but cannot establish the superiority of any management strategy.

## 1. Introduction

Renal dysfunction after endovascular aneurysm repair (EVAR) is usually multifactorial and may reflect contrast exposure, hemodynamic instability, microembolization, or progression of pre-existing chronic kidney disease. Direct renal artery compromise is a distinct entity because it may be immediately reversible but can also result in irreversible renal loss, severe acute kidney injury (AKI), renovascular hypertension, or dialysis [1,2,3].

After standard infrarenal EVAR, renal perfusion may be impaired by inadvertent fabric coverage of the renal ostium, interaction between the ostium and suprarenal fixation struts, proximal cuff deployment, endograft migration, thrombosis, or atheroembolization from a hostile proximal aorta. Although these events are frequently grouped together, they differ substantially in accessibility, probability of successful recanalization, and expected renal recovery.

Published evidence is mainly limited to isolated reports and small case series. Renal function has recovered after both immediate and delayed revascularization, indicating that elapsed time should not be the only determinant of salvage. Residual nephrogram, cortical enhancement, collateral perfusion, kidney size and baseline renal reserve may all identify patients who can benefit from intervention [4,5,6].

The present study retrospectively reviewed a consecutive cohort of standard EVAR procedures to estimate the incidence of direct renal artery compromise and to describe patient-level mechanisms, management decisions, renal-function trajectories, target-vessel outcomes, and long-term survival. We further sought to determine whether a mechanism-guided framework could support individualized renal salvage.

## 2. Materials and Methods

### 2.1. Study Design, Setting, and Eligibility

This retrospective, single-center observational cohort included all consecutive patients undergoing elective standard EVAR for an infrarenal abdominal aortic aneurysm between March 2016 and June 2025. Standard EVAR was defined as implantation of an off-the-shelf infrarenal aorto-bi-iliac endograft without planned renal fenestrations, branches, chimneys, periscopes, or other renal incorporation. A renal stent placed as an unplanned bailout maneuver did not reclassify the index procedure as complex EVAR. Concomitant endovascular treatment of iliac aneurysmal disease was permitted. The study size was determined by all eligible procedures performed during the prespecified study period; no formal sample-size calculation was performed.

Patients with pararenal or thoracoabdominal aneurysms, fenestrated or branched repair involving the renal arteries, planned renal parallel grafting, pre-existing complete occlusion of the affected renal artery, or urgent or emergent presentation were excluded.

To reduce selection and ascertainment bias, the entire institutional standard EVAR registry was screened. Candidate renal events were cross-checked against procedural reports, discharge documentation, and available perioperative imaging; no sampling was performed.

One renal event occurring in 2016 (Case 1) had been included in a previous report from our group addressing technical intraoperative complications after standard EVAR [1]. The present renal-specific analysis uses the complete 2016–2025 standard-EVAR denominator, includes two additional renal-compromise phenotypes, and provides extended renal and survival follow-up.

### 2.2. Data Sources, Outcomes, and Definitions

Demographics, cardiovascular comorbidities, aneurysm morphology, endograft type, adjunctive procedures, event laterality, presumed mechanism, time of recognition, rescue technique, technical success, serum creatinine, estimated glomerular filtration rate (eGFR), dialysis, imaging follow-up, renal reintervention, and survival were extracted from the dedicated renal-event dataset and the institutional EVAR registry. Follow-up duration was calculated from the index EVAR to the most recent clinical assessment or death.

The primary outcome, direct renal artery compromise, included complete occlusion or severe ostial compromise/subocclusion. Complete occlusion was defined as absence of antegrade flow in a main renal artery on angiography, computed tomography angiography, or duplex ultrasound.

Severe non-occlusive renal artery compromise was identified when angiography or CTA showed markedly reduced ostial patency or antegrade renal perfusion, interpreted together with the clinical and biochemical findings.

Technical success was defined as angiographic restoration of antegrade renal perfusion after the intended rescue procedure. AKI stage was assigned from the highest available inpatient creatinine according to Kidney Disease: Improving Global Outcomes criteria [7]. eGFR was calculated with the 2021 CKD-EPI creatinine equation [8].

### 2.3. Statistical Analysis

Continuous variables were summarized as mean ± standard deviation or median with interquartile range (or range for the three event cases), as appropriate. Categorical variables were reported as counts and percentages. The incidence of direct renal artery compromise was calculated using the number of affected patients as the numerator and all eligible standard EVAR procedures as the denominator; an exact binomial 95% confidence interval was reported. Analyses were performed using IBM SPSS Statistics version 21.0 (IBM Corp., Armonk, NY, USA).

The study is reported in accordance with the Strengthening the Reporting of Observational Studies in Epidemiology (STROBE) recommendations (Supplementary Materials) [9].

## 3. Results

### 3.1. Overall Cohort and Incidence

Between March 2016 and June 2025, 973 eligible elective standard EVAR procedures were performed. Three patients developed direct renal artery occlusion or severe compromise, corresponding to an observed incidence of 0.31% (exact 95% confidence interval, 0.06–0.90%). Median age among affected patients was 78 years (range, 64–79 years); two were men, and one had known chronic kidney disease. The events represented three phenotypes: immediate mechanical ostial coverage, atherothrombotic occlusion associated with a thrombus-bearing proximal aorta, and bilateral renal compromise after proximal cuff deployment (Table 1).

Endovascular renal salvage was attempted in two patients and was technically successful in both. The patient with unilateral embolic occlusion was managed without renal revascularization. All three patients developed AKI (KDIGO stage 1 in one and stage 2 in two), but none required temporary or permanent dialysis. Median clinical follow-up was 67.6 months (range, 7.4–120.4 months). Two patients were alive at the latest follow-up, whereas one died more than 5 years after the index procedure (Table 2).

### 3.2. Case 1—Immediate Right Renal Coverage and Trans-Free-Flow Stenting

**Figure 1:**
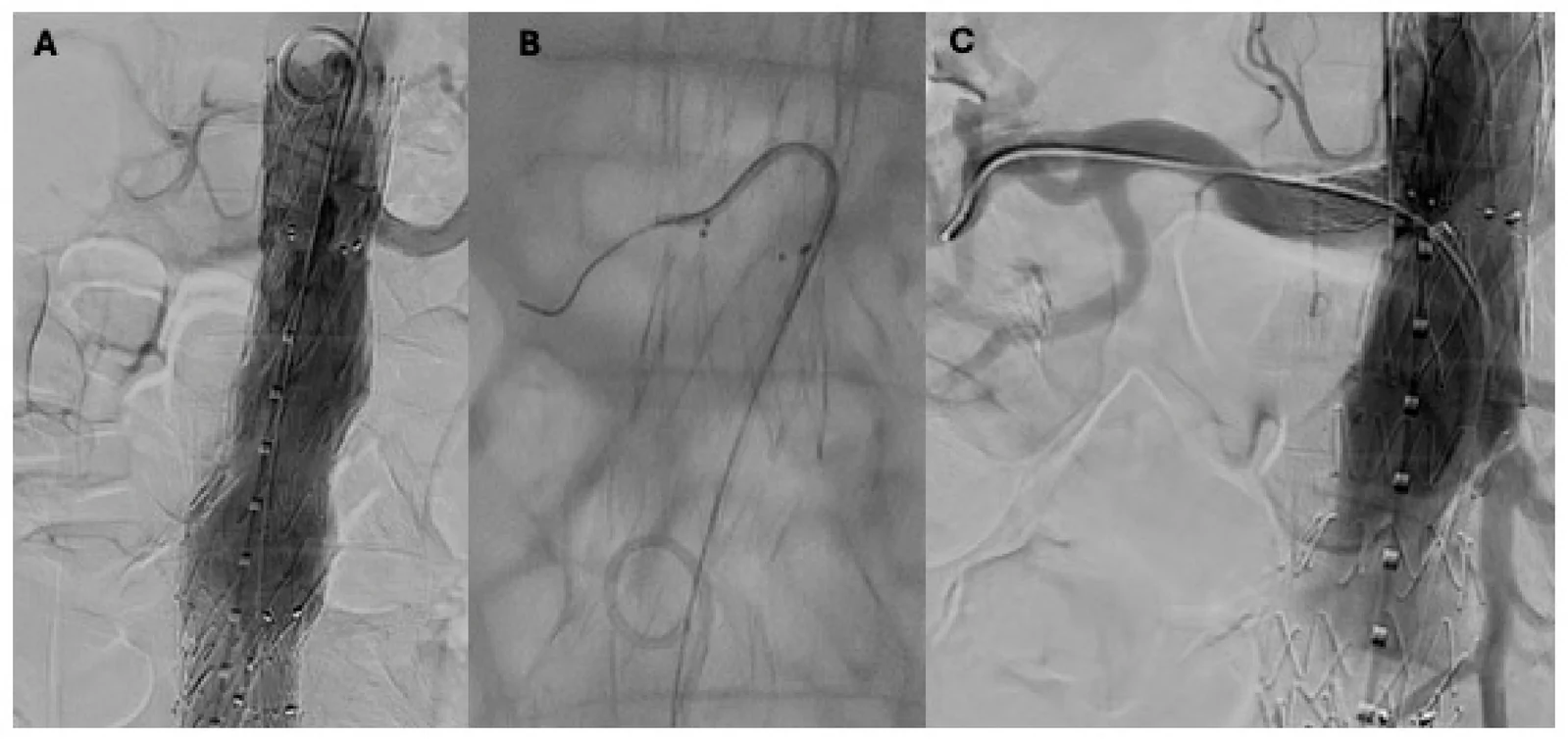
Immediate right renal ostial coverage and trans-free-flow stenting. (**A**) Completion angiography showing impaired right renal perfusion after endograft deployment. (**B**) Catheterization through the interstices of the suprarenal free-flow stent. (**C**) Completion angiography after balloon-expandable renal stenting showing restored perfusion.

A 64-year-old man underwent elective EVAR in 2016 using a Cook Zenith Alpha (Cook Medical, William Cook Europe ApS, Bjæverskov, Denmark) aorto-bi-iliac endograft with suprarenal fixation and a 28 mm proximal component. The right renal ostium was inadvertently covered during deployment. The event was recognized on completion angiography, and the renal artery was catheterized from an upper-extremity access through the interstices of the free-flow stent. A Visi-Pro balloon-expandable renal stent was deployed, producing immediate restoration of renal perfusion (Figure 1).

Serum creatinine increased from 0.50 mg/dL before EVAR to 1.19 mg/dL at discharge on postoperative day 4, corresponding to an eGFR decrease from approximately 114 to 68 mL/min/1.73 m^2^ and KDIGO stage 2 AKI. Subsequent duplex surveillance confirmed renal-stent patency, and the patient remained independent of renal replacement therapy throughout 120.4 months of follow-up.

### 3.3. Case 2—Atherothrombotic Right Renal Occlusion Managed Conservatively

A 79-year-old man with chronic kidney disease underwent elective EVAR in 2018. Preoperative imaging showed a 55 mm infrarenal aneurysm and a proximal neck measuring 27 mm in diameter and 20 mm in length, with 75-degree angulation and approximately 40% circumferential mural thrombus adjacent to the renal ostia. A Gore Excluder C3 endograft with a 35 mm proximal component was implanted; associated iliac aneurysmal disease was treated with an iliac branch device. During or shortly after EVAR, right renal artery occlusion was attributed to displacement of atherothrombotic material from the hostile proximal aorta.

**Figure 2:**
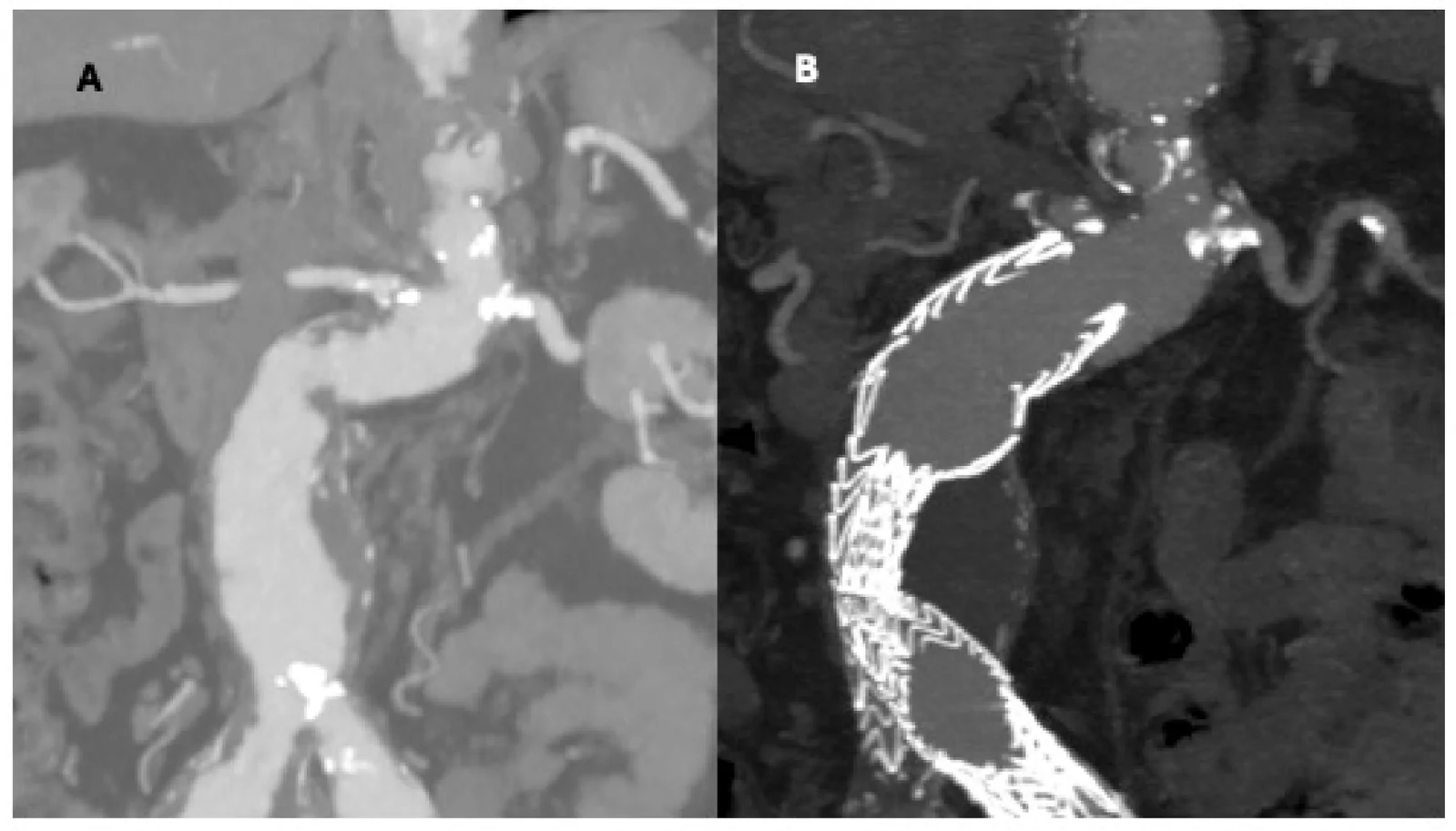
Atherothrombotic right renal artery occlusion managed conservatively. (**A**) Preoperative computed tomography angiography demonstrating a thrombus-bearing proximal aortic neck close to the renal ostia. (**B**) Postoperative imaging showing right renal artery occlusion with preserved perfusion of the contralateral kidney.

Because the occlusion was unilateral, contralateral renal function was preserved, and further catheter manipulation within the thrombus-bearing proximal aorta was considered to carry a substantial embolic risk, renal revascularization was not attempted (Figure 2).

Serum creatinine increased from 1.49 mg/dL (eGFR approximately 47 mL/min/1.73 m^2^) to a documented peak of approximately 3.00 mg/dL (eGFR approximately 22 mL/min/1.73 m^2^), then decreased to 2.50 mg/dL (eGFR approximately 26 mL/min/1.73 m^2^) by discharge on postoperative day 9, corresponding to KDIGO stage 2 AKI. Dialysis was not required. The patient died after 67.6 months of follow-up; the late death was unrelated to the renal event or index aortic repair.

The decision to avoid further aortic instrumentation was made in the setting of unilateral renal artery occlusion, preserved perfusion of the contralateral kidney, pre-existing chronic kidney disease, and substantial thrombus near the renal ostia. The patient’s subsequent rise in serum creatinine from 1.49 to approximately 3.00 mg/dL underscores that observation did not imply absence of clinically meaningful renal injury.

### 3.4. Case 3—Bilateral Renal Compromise After Proximal Cuff Deployment

A 78-year-old woman underwent elective EVAR in 2025 for a 64 mm infrarenal aneurysm. The proximal neck measured 21 mm in diameter and 11 mm in length, with approximately 22% mural thrombus. A Gore Excluder Conformable aorto-bi-iliac endograft was implanted. A proximal cuff was added to optimize sealing immediately below the renal arteries; deployment produced partial coverage of the left renal ostium.

Computed tomography angiography on postoperative day 2 demonstrated complete left renal artery occlusion and severe right renal ostial compromise. Because the left renal ostium was not considered safely accessible and the right renal artery retained a treatable channel, the right side was selected for salvage.

**Figure 3:**
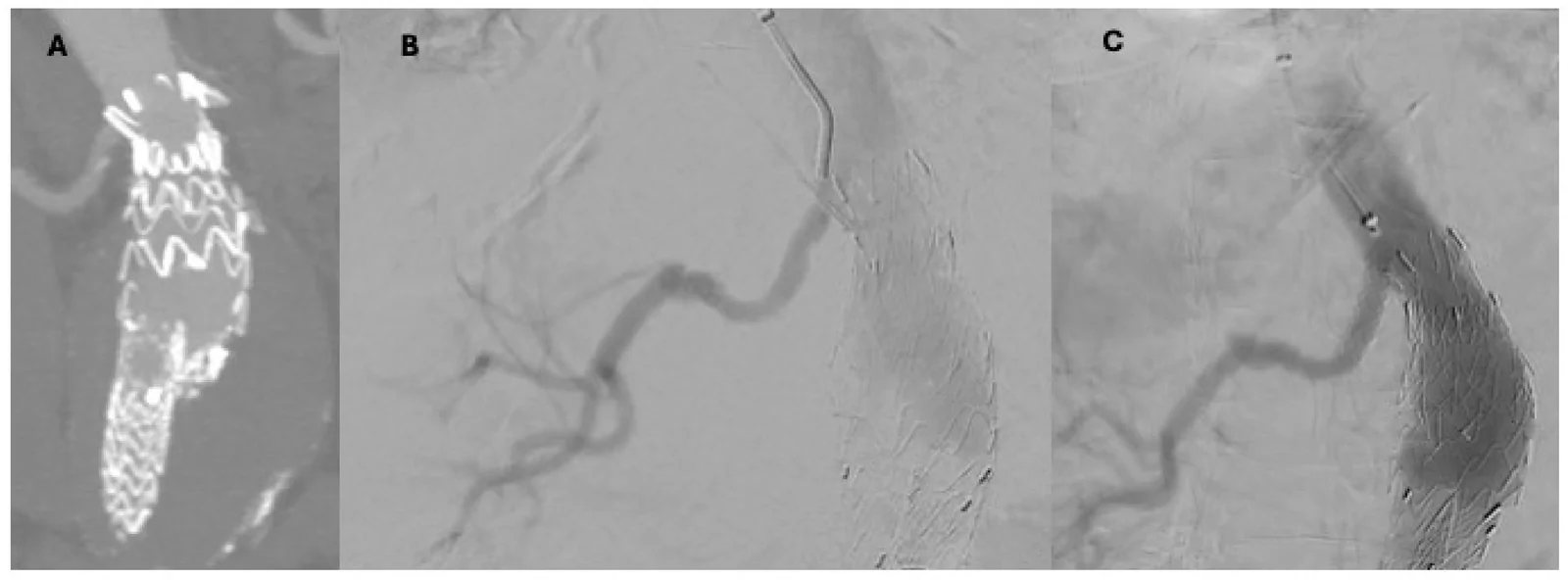
Bilateral renal compromise after proximal cuff deployment and selective right renal salvage. (**A**) Early postoperative computed tomography angiography showing complete left renal artery occlusion and severe right ostial compromise. (**B**) Selective right renal angiography before stenting. (**C**) Completion angiography after parallel covered-stent placement showing restored right renal perfusion.

On postoperative day 2, the right renal artery was treated with a parallel Advanta V12 covered stent, with angiographic restoration of perfusion. Serum creatinine increased from 0.65 mg/dL before EVAR to 1.16 mg/dL at discharge on postoperative day 9; eGFR decreased from approximately 90 to 48 mL/min/1.73 m^2^, corresponding to KDIGO stage 1 AKI. Dialysis was not required. At 7.4 months, the patient was alive, and aneurysm diameter had decreased from 64 to 60 mm (Figure 3).

Duplex ultrasound at approximately 6 months confirmed right renal-stent patency and stable renal function without renal replacement therapy.

## 4. Discussion

In this retrospective cohort of 973 elective standard EVAR procedures, direct renal artery compromise occurred in three patients (0.31%) and comprised three mechanistically distinct patterns. The first was an immediate mechanical event amenable to catheterization through the free-flow segment of a suprarenal fixation device. The second was an embolic occlusion arising from a hostile, thrombus-rich aorta and was managed without further renal intervention. The third combined complete occlusion on one side with severe contralateral compromise after proximal cuff deployment and required selective salvage of the kidney with the greatest technical and functional potential. These patterns support mechanism-guided rather than uniform management.

The management decisions in this series are illustrative rather than comparative: three heterogeneous events cannot determine whether early revascularization, delayed rescue, or observation should be preferred. Our proposed approach represents clinical considerations suggested by these cases and the available literature, not a validated treatment algorithm.

Direct renal artery compromise was uncommon in our cohort, with an observed incidence of 0.31% and a wide exact 95% confidence interval of 0.06–0.90%. Greenberg et al. reported renal occlusion in approximately 1% of patients treated with a suprarenal fixation device, while a meta-analysis of 21 nonrandomized studies found no clear difference in renal artery occlusion or dialysis between suprarenal and infrarenal fixation [2,3]. Comparisons should remain cautious because definitions, device generations, and postoperative imaging protocols differ across studies.

Recognition must be rapid but should extend beyond the completion angiogram. Partial ostial coverage, dynamic impingement, or evolving thrombus may initially preserve some renal perfusion. An unexpected rise in creatinine, oliguria, new or severe hypertension, flank pain, or metabolic deterioration should prompt targeted renal imaging. Conversely, unilateral renal loss may be clinically silent when the contralateral kidney is preserved. Direct assessment of both renal arteries is therefore important even when the biochemical signal is modest.

The observed incidence refers to recognized, clinically significant direct renal artery events rather than all anatomical renal artery occlusions. Ascertainment depended on recorded procedural and clinical findings and available imaging; nonuniform postoperative surveillance may have missed clinically silent unilateral or transient events. Accordingly, comparisons with historical studies using different definitions and surveillance schedules should be made cautiously.

The therapeutic window is not determined by time alone. Hedayati et al. described functional recovery after renal stenting despite more than 48 h of occlusion [4]. Hamish et al. and Adu et al. reported renal recovery after delayed renal revascularization, including patients who had already started dialysis [5,6]. Persistent cortical enhancement, residual nephrogram, collateral perfusion, preserved kidney size, incomplete occlusion, and bilateral or solitary-kidney involvement may justify an attempt at salvage after delayed recognition.

When the renal ostium can be reached, an endovascular-first approach minimizes the physiological burden of reintervention. Options include cannulation through a suprarenal free-flow stent, passage between the endograft and aortic wall, balloon angioplasty, balloon-expandable renal stenting, parallel covered stenting, aspiration or thrombectomy for selected thrombotic lesions, and endograft relining. Device choice should reflect whether the problem is fabric coverage, external compression, ostial dissection, or thromboembolism and should also account for the proximal seal and risk of type Ia endoleak.

Our cases suggest a structured approach to individualized assessment rather than a validated treatment algorithm. Procedural angiography and preoperative CTA first help distinguish mechanical ostial coverage from suspected thromboembolism. Laterality, contralateral renal function, baseline chronic kidney disease, and solitary-kidney status inform the potential consequences of nonintervention. When available, residual cortical enhancement, a nephrogram, collateral perfusion, and preserved kidney size can contribute to assessment of viability. Ostial accessibility and the possible harms of repeat intervention—including additional embolization from a thrombus-bearing aorta, contrast exposure, and compromise of the proximal seal—should then be weighed with the renal risk of observation. These considerations are not ranked or validated by the present cohort and require clinical judgment.

Recent reports have described catheter-based rescue following inadvertent renal artery coverage during EVAR and the midterm outcomes of bailout renal stenting in contemporary practice [10,11]. These studies support the technical feasibility of selected rescue strategies; Pastor et al. [11] reported an incidence of 1% (7 cases out of a total of 748 EVARs), considering the renal ostium coverage due to technical endograft implantation.

The second patient emphasizes that not every unilateral occlusion should be treated. In a patient with a functioning contralateral kidney and a high risk of further embolization, the procedural burden of recanalization may outweigh the expected gain from restoring one kidney. By contrast, the third patient had bilateral compromise, so preservation of at least one renal artery was essential. Selective right-sided rescue converted a potentially dialysis-producing event into a unilateral renal loss with preserved global renal function. This individualized balance among laterality, renal reserve, viability, accessibility, and risk is the central personalized-medicine contribution of the series.

All three patients developed AKI, although none had permanent dialysis recorded. Serum creatinine rose more than twofold in the first two patients and by approximately 78% in the third. These trajectories demonstrate why technical success alone is insufficient: reporting should include peak and discharge creatinine, eGFR, dialysis, blood-pressure response, target-vessel patency, renal reintervention, and survival. Serum creatinine may still underestimate unilateral functional loss, reinforcing the value of longitudinal imaging.

Several limitations should be acknowledged. This was a single-center retrospective cohort with only three events. Although the study provides an observed incidence, the confidence interval is wide, and the small event count precludes identification of predictors or comparison of management strategies. Event ascertainment depended on registry data, clinical documentation, and available imaging, so transient or clinically silent compromise may have been missed. Laboratory and imaging follow-up were not performed at uniform intervals, and split renal function was not systematically measured. Finally, serum creatinine is an imperfect marker of unilateral renal injury. Nevertheless, the series includes mechanical and embolic mechanisms, intervention and observation, and follow-up extending beyond 10 years.

## 5. Conclusions

Recognized, clinically significant direct renal artery compromise occurred in 3 of 973 elective standard EVAR procedures (0.31%); clinically silent events may have been missed. The three cases illustrate distinct mechanisms and different management decisions but cannot determine which strategy is preferable. Mechanism, laterality, renal reserve, evidence of residual viability, ostial accessibility, and procedural risk may inform individualized assessment. Larger studies with standardized imaging and longitudinal renal evaluation are needed.

## Figures and Tables

**Table 1:** Baseline characteristics, anatomy, and index EVAR details.

Variable	Case 1	Case 2	Case 3
Age, sex	64 years, male	79 years, male	78 years, female
Presentation	Elective, unruptured	Elective, unruptured	Elective, unruptured
ASA class	III	III	IV
Baseline CKD	No	Yes	No
Baseline creatinine; eGFR	0.50 mg/dL; 114	1.49 mg/dL; 47	0.65 mg/dL; 90
Index device	Cook Zenith Alpha; suprarenal fixation	Gore Excluder C3; iliac branch adjunct	Gore Excluder Conformable plus proximal cuff

CKD, chronic kidney disease; eGFR, estimated glomerular filtration rate in mL/min/1.73 m^2^, calculated using the 2021 CKD-EPI creatinine equation.

**Table 2:** Renal event, management, and temporal evolution.

Variable	Case 1	Case 2	Case 3
Affected artery	Right occlusion	Right occlusion	Left occlusion plus severe right compromise
Detection	Completion angiography	Intraoperative/early postoperative	CTA on postoperative day 2
Mechanism	Inadvertent ostial coverage/impingement	Atherothrombotic embolization from shaggy aorta	Proximal cuff-related ostial coverage/impingement
Management	Immediate trans-free-flow renal stent (Visi-Pro balloon expandable stent)	Conservative management	Parallel right renal stent graft (Advanta V12) on postoperative day 2
Technical success	Yes	Not applicable	Yes
Creatinine: baseline/peak/discharge	0.50/1.19/1.19 mg/dL	1.49/3.00/2.50 mg/dL	0.65/1.16/1.16 mg/dL
AKI/dialysis	KDIGO 2/none	KDIGO 2/none	KDIGO 1/none
Follow-up, creatinine	120.4 months; alive; 1.35 mg/dL	67.6 months; late death; 3.00 mg/dL	7.4 months; alive; 1.1 mg/dL
Renal outcome	Patent rescued artery; dialysis-free	Dialysis-free with unilateral renal loss	Patent right renal stent; dialysis-free

AKI, acute kidney injury; CTA, computed tomography angiography.

## Data Availability

The data presented in this study are available on request from the corresponding author due to privacy reasons.

## Supplementary Materials

The following supporting information can be downloaded at: https://www.mdpi.com/article/10.3390/jpm16100519/s1, File S1: STROBE Cohort Checklist.
