# Supplementary material for: Renal Artery Occlusion After Standard Endovascular Aneurysm Repair: Incidence, Mechanisms, and Individualized Management
Source: J Pers Med. 2026 Oct 5;16(10):519. doi: 10.3390/jpm16100519 (PMC13706477; doi:10.3390/jpm16100519)
Supplement: Supplementary file 1 [file jpm-16-00519-s001.zip › jpm-4560694-supplementary.pdf]

STROBE Cohort Checklist

Completed checklist for the submitted retrospective cohort study

Manuscript: Renal Artery Occlusion after Standard Endovascular Aneurysm Repair: Incidence, Mechanisms, and Individualized Management

Item	Reporting requirement	Location in manuscript
1a	Identify the observational design in the title or abstract.	Structured abstract (Methods)
1b	Provide a balanced abstract summary of methods and findings.	Structured abstract
2	Explain the scientific background and rationale.	1. Introduction, paragraphs 1-3
3	State the objectives or hypotheses.	1. Introduction, final paragraph
4	Present the key design elements early.	2.1. Study Design, Setting, and Eligibility
5	Describe setting, location, and relevant study and follow-up dates.	2.1; 3.1; individual case timelines
6a	Give eligibility criteria and participant selection/follow-up methods.	2.1; 2.2
6b	Report matching criteria if applicable.	Not applicable; no matched design
7	Define outcomes, exposures, predictors, confounders, and diagnostic criteria.	2.2. Data Sources, Outcomes, and Definitions
8	Describe data sources and measurement methods.	2.2; Tables 1-2
9	Describe efforts to address potential bias.	2.1, registry-screening paragraph; 4. Discussion, limitations
10	Explain how study size was determined.	2.1, all eligible EVAR during the study period
11	Explain handling of quantitative variables.	2.3. Statistical Analysis
12a	Describe statistical methods.	2.3. Statistical Analysis
12b	Describe subgroup or interaction analyses.	Not performed because only three events occurred
12c	Explain handling of missing data.	2.3; no imputation, available patient-level observations
12d	Explain handling of loss to follow-up.	Follow-up reported to last assessment or death; 2.2 and Table 2
12e	Describe sensitivity analyses.	Not applicable
13a	Report participant numbers at each study stage.	3.1: 973 eligible procedures; three events
13b	Give reasons for non-participation or exclusion.	2.1 eligibility and exclusions
13c	Consider a flow diagram.	Not necessary for a registry-wide denominator with no sampling; optional

Item	Reporting requirement	Location in manuscript
14a	Report participant characteristics and relevant clinical data.	3.1; Table 1
14b	Indicate missing data for variables of interest.	2.3; case-level reporting in Tables 1-2
14c	Summarize follow-up time.	3.1; Table 2
15	Report numbers of outcome events over time.	3.1; Cases 1-3; Table 2
16a	Report estimates with precision.	Abstract; 3.1: 0.31%, exact 95% CI 0.06%-0.90%
16b	Report category boundaries when continuous variables are categorized.	AKI categories defined by KDIGO; 2.2
16c	Where relevant, translate relative estimates into absolute risk.	Absolute incidence reported; no relative estimates
17	Report other analyses, including subgroup or sensitivity analyses.	None performed
18	Summarize key results with reference to objectives.	4. Discussion, opening paragraph
19	Discuss limitations and potential bias or imprecision.	4. Discussion, limitations paragraph
20	Provide a cautious interpretation in context.	4. Discussion; 5. Conclusions
21	Discuss generalizability.	4. Discussion, single-center and event-rarity limitations
22	State funding source and funder role.	Funding statement

Source: STROBE Statement cohort checklist (https://www.strobe-statement.org/checklists/).
